# Surface Projection of Interosseous Foramen of the Leg: Cadaver Study

**DOI:** 10.1155/2016/6312027

**Published:** 2016-11-13

**Authors:** Eric Arguello, Carissa Stoddard, Hao (Howe) Liu, Mike Richardson, Andrea Hartis

**Affiliations:** Department of Physical Therapy, University of North Texas Health Science Center, 3500 Camp Bowie Blvd., Fort Worth, TX 76107, USA

## Abstract

*Purpose*. This study was conducted to identify the surface projection of the interosseous foramen and associated structures of the proximal leg using the average clinician's thumb width as a quick measurement to assist in differential diagnosis and treatment.* Methods*. Twelve cadavers (5 males and 7 females, age range = 51–91 years, and mean age = 76.9) were dissected for analysis. Location and size of interosseous foramen, location of anterior tibial artery, location of deep fibular nerve, and corresponding arterial branches were measured and converted into thumb widths.* Results*. Mean thumb width measured among the cadavers was 17.94 ± 3.9 mm. The interosseous foramen measured was approximately 1 thumb width vertically (18.47 ± 3.0 mm) and 1/2 thumb width horizontally (7.32 ± 2.1 mm) and was located approximately 1 thumb width distally to the tibial tuberosity (20.81 ± 6.8 mm) and 2 thumb widths (37.47 ± 4.7 mm) lateral to the tibial ridge. The anterior tibial artery and deep fibular nerve converged approximately 4 thumb widths (74.31 ± 14.8 mm) inferior to the tibial tuberosity and 2 thumb widths (33.46 ± 4.9 mm) lateral to the tibial ridge.* Conclusion*. Clinicians may identify anatomical structures of the proximal leg with palpation using the thumb width for measurement.

## 1. Introduction

Physical therapists and other clinicians use palpation to locate anatomical structures for evaluation and treatment [1–4]. Accurate palpation is important during the examination in order to determine a differential diagnosis and identify structures requiring treatment [1, 5]. Although not standardized, clinicians frequently use thumb width as a measurement tool to approximate the location of anatomical structures from bony landmarks [6–9].

Physical therapists [6], physicians [7], and acupuncturists [9] use the thumb as a quick and economic measurement tool in clinical practice. Physical therapists use palpation and thumb width for measurement to define the area to be treated, for provocation of structures, and to assess joint mobility [6, 7]. Physicians use the thumb width as a measurement tool to locate the correct anatomical injection sites for treatments [7]. Acupuncturists use thumb width as a measurement tool to locate acupuncture points for needle placement [9].

Anterior leg pain related to chronic exertional compartment syndrome (CECS) is commonly managed in clinical practice. Accurate palpation to identify structures of the lower extremity (LE) is important for differential diagnosis and treatment of CECS. The anterior compartment of the leg is a relatively closed space surrounded by muscular septum, interosseous membrane (IOM), and the tibia and fibula bones [10]. The anterior compartment of the leg is located between the tibial tuberosity and fibular head and is occupied with the extensor digitorum, tibialis anterior, and fibularis tertius muscles while its inferior region is contained by the superior and inferior extensor retinacula across the medial and lateral malleoli.

The IOM of the leg is a sheet of connective tissue that bridges the distance between the tibial and fibular shafts. There are two foramens in the IOM, one at the proximal end and one at the distal end, for vessels to pass between the anterior and posterior compartments [10].

As pressure increases, anatomical weak spots within the compartment will absorb the pressure; one of the probable weak spots is the proximal interosseous foramen (IOF) where the anterior tibial artery (ATA) and anterior tibial vein (ATV) traverse. Increased compression of the IOF, ATA, and ATV will decrease the oxygen supply to the anterior compartment and decrease the rate of venous return.

In order to make an accurate differential diagnosis of CECS and apply treatment, it is important for clinicians to quickly and accurately locate the IOF and associated structures during the clinical examination [11, 12]. Previous studies using cadaver dissections of the leg have been performed to identify the relationship of the ATA to the tibia [13] and to measure the length and width of the IOF for surgical interventions [14]. However, no studies have been performed to identify the surface projection of the IOF and ATA in the proximal leg with palpation.

Liu et al. [15] performed cadaver dissections to calculate thumb interphalangeal joint (IPJ) width coefficients to improve the accuracy of palpation of structures in the upper extremity (UE). Anatomical measurements of structures in the UE were converted into thumb widths (TWs) for quick and accurate identification during the clinical examination. Therefore, the purpose of this study was to identify the surface projection of the IOF and ATA and associated structures using thumb width as the method of measurement to assist in differential diagnosis and treatment of CECS in the lower extremity.

## 2. Methods

This study was qualified for exemption from the institutional review board approval process. Twenty-four lower extremities (LEs) from 12 Caucasian adult cadavers (5 males and 7 females, age range = 51–91 years, and mean age = 76.9) were used in this study.

Each cadaver's LEs had previously been dissected and the researchers had only to dissect any remaining tissue to expose the IOF and ATA under the extensor digitorum and tibialis anterior muscles. All IPJ TWs of the cadavers, location and size of IOF, location of ATA and ATV, location of deep fibular nerve (DFN), and corresponding arterial branches were measured and analyzed in the proximal region of the anterior compartment of the leg. Dissecting instruments included surgical scalpels, scissors, forceps, a digital caliper, and a transparent right-angled ruler.

In the dissection, measurements were performed on 12 cadavers to denote the locations of the IOF and ATA under the extensor digitorum and tibialis anterior muscles in relation to the transverse and vertical planes through the tibial tuberosity. During measurement, both transverse and vertical planes were maintained using a transparent ruler with it placed on the most prominent part of the tibial tuberosity (TT). The distances (in millimeters) from the midpoint of the IOF to the transverse and the vertical planes through TT were measured with a digital caliper with the extremity being measured in full-knee extension.

Specific distances measured included the vertical distance from the most prominent portion of the TT aligned with a ruler to the midpoint of the IOF, in addition to the horizontal distances from the anterior edge of the tibia to the midpoint of the IOF. In addition, the vertical length and horizontal width of the IOF were measured. The length of the ATA was measured as the linear distance from the midpoint of the IOF to its convergence with the DFN. The length of the tibia was also measured. During measurement, all distances were recorded with one researcher attending to all the written recordings and one researcher performing all the dissections. Once two measurements (in millimeters) were obtained for a specific distance (i.e., IOF length), the average value was calculated. In addition to these measurements, the IPJ TWs with skin and subcutaneous tissue intact around the joint from all 12 cadaver upper extremities (UEs) and from the dominant hands of a convenience sample of 43 healthy adults (physical therapy students) were measured. Each of these IPJ TWs was measured twice, and the mean of the two measurements was calculated. To ensure in vivo carryover of the data the average value of IPJ TW from the healthy adults was compared with the average value of IPJ TW from the cadavers. Values from both the cadaver and in vivo IPJ TW measurements were combined to calculate an overall average. This overall average was used to describe IOF dimensions, IOF location in the vertical and horizontal planes in relationship to the TT, and length of the ATA from the IOF to the DFN convergence point. For clinical purposes, average values of all distances measured by the caliper in this study were converted into numbers of IPJ TWs.

## 3. Data Analysis

The IPJ TWs of cadavers and healthy adults were compared for differences using unpaired Student's *t*-test. Difference in gender was also tested by unpaired Student's *t*-test. Statistical significance was defined as *P* < 0.05. IBM SPSS for Windows 19.0 software (New York, NY) was used for all analyses.

## 4. Results

**Figure 1:**
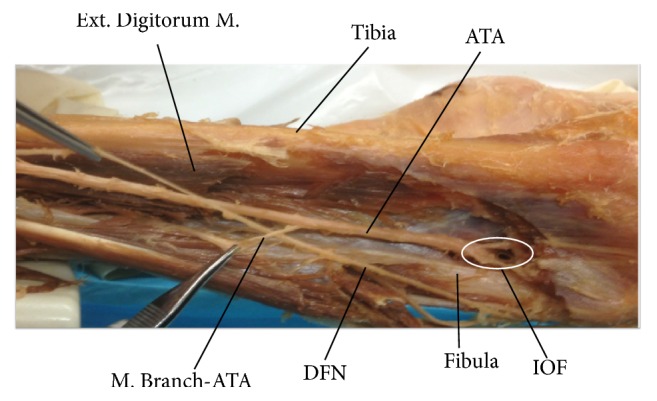
Dissection of the anterior compartment of the leg showing the interosseous foramen (IOF) and anterior tibial artery (ATA). Ext. Digitorum M.: extensor digitorum muscle; M. Branch-ATA: muscular branch of the anterior tibial artery; and DFN: deep femoral nerve.

**Figure 2:**
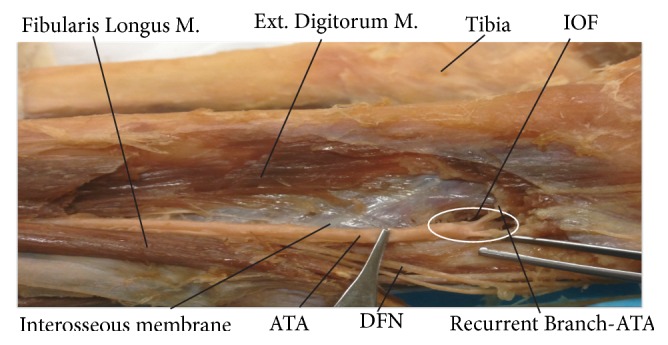
Dissection of the anterior compartment of the leg showing the interosseous membrane and interosseous foramen (IOF). Fibularis Longus M.: fibularis longus muscle; Ext. Digitorum M: extensor digitorum muscle; ATA: anterior tibial artery; and Recurrent Branch-ATA: recurrent branch of anterior tibial artery.

**Figure 3:**
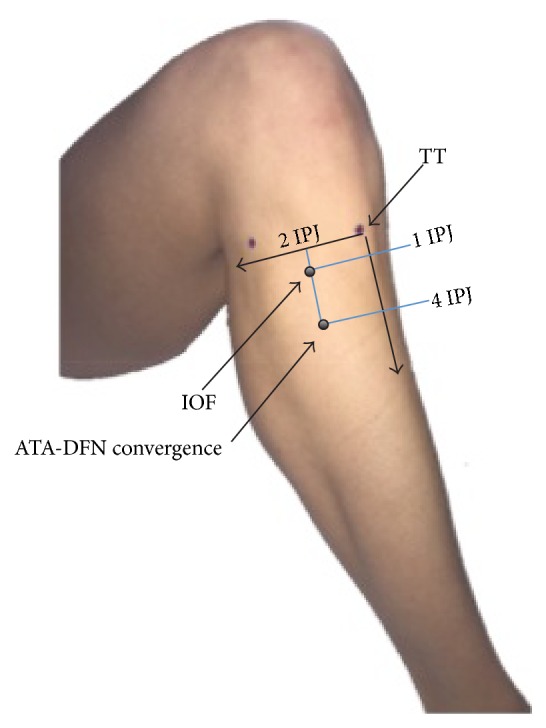
Surface anatomy of the interosseous foramen and anterior tibial artery-deep femoral nerve convergence point (anterolateral view) in knee flexion. IOF: interosseous foramen. IPJ: interphalangeal joint width of thumb. TT: tibial tuberosity.

**Figure 4:**
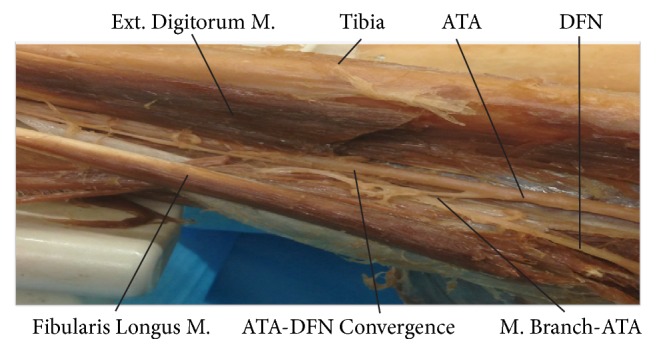
Dissection of the anterior compartment of the leg showing the anterior tibial artery and deep fibular nerve convergence point (ATA-DFN Convergence). Ext. Digitorum M.: extensor digitorum muscle; ATA: anterior tibial artery; DFN: deep fibular nerve; Fibularis Longus M.: fibularis longus muscle; and M. Branch-ATA: muscular branch of the anterior tibial artery.

No significant differences were found between genders for all measurements. The average IPJ TW from all cadavers was 17.94 ± 3.9 mm while the average IPJ TW from 43 healthy physical therapy students was 19.13 ± 5 mm. No significant difference of the IPJ TW was identified between the cadavers and the healthy subjects. The IOF was measured in an oval shape vertically with its diameter approximately 1 TW vertically (18.47 ± 3.0 mm) and 1/2 TW horizontally (7.32 ± 2.1 mm) (Figures 1 and 2). The center point of the IOF was located approximately 1 TW (20.81 ± 6.8 mm) distal from the TT and 2 TW (37.47 ± 4.7 mm) lateral from the tibial ridge (Figure 3). The ATA and ATV were bundled together to pass through the IOF and descend inferiorly for approximately 3 TWs (53.77 ± 11.9 mm) along the anterior surface of the IOM converging with the DFN (Figure 4). The convergence was approximately 4 TWs (74.31 ± 14.8 mm) distal to the tibial tuberosity and 2 TWs (33.46 ± 4.9 mm) lateral to the tibial ridge (Figure 3). Before the convergence, the ATA divided into 1 (54.2%), 2 (33.3%), or 3 (12.5%) branches to the extensor digitorum longus and tibialis anterior muscles. The average measured length of the tibia was 33.51 ± 9.2 cm.

## 5. Discussion

Our anatomical study focused on the location and size of the IOF, location of the proximal ATA and DFN, and corresponding arterial branches due to their possible involvement in CECS. Increased pressure in the anterior compartment of the leg with CECS may compress the above anatomical structures leading to reduced blood flow and/or nerve damage. Therefore, it is important for the clinician to easily locate these structures for evaluation and treatment.

The results of the current study are similar to those of Ebraheim et al. [13] that reported the IOF measured in vertical length was 17.1 ± 4.1 mm and 8.9 ± 1.1 mm in horizontal width. In our study we used the tibial tuberosity as the reference anatomical landmark to make our measurements in order to determine the location of the IOF and ATA in the vertical and horizontal planes in the proximal portion of the leg with palpation. In contrast, the studies by Ebraheim et al. [13] and Heidari et al. [14] used the tibial plateau and head of the fibula as reference anatomical landmarks to determine the location of the IOF in the vertical plane during surgical interventions. Ebraheim et al. [13] reported the average length of the tibia in their study was 30.1 ± 2.2 cm. In our study the average measured length of the tibia was 33.51 ± 9.2 cm. This may reflect the difference in stature between the cadavers measured in each study.

No significant difference between genders was found in our study. However, it is known that gender, race, and age result in variation of anatomic measurements [16–18]. The cadavers analyzed in this study were older Caucasian adults (51–91 years, mean age = 76.9). Therefore, a limitation of this study is that the results may only be applicable to individuals in this age range and this race. Future studies should include cadavers with a variety of races and ages.

We did not find a statistically significant difference in IPJ width between the cadaver group and the healthy adult group. Therefore, the location of the IOF and ATA can be approximated using the clinician's own thumb IPJ width during clinical examination and treatment. However, a precise and more repeatable measurement tool would be required for the surgeon when operating.

## 6. Conclusion

Based on the current study, a clinician should be able to approximate the location of the IOF and ATA in the proximal leg at one thumb IPJ width perpendicular to the transverse plane through the TT and two thumb IPJ widths perpendicular to the vertical plane through the TT. Similarly, the location of the ATA and DFN convergence point under the tibialis anterior is approximately 4 thumb IPJ widths perpendicular to the transverse plane through the TT and 2 thumb IPJ widths perpendicular to the vertical plane through the TT. Future studies should validate the clinical effectiveness of this method of measurement with palpation in a patient population. Based on this study, clinicians may use TW to identify the size and location of the IOF and ATA and corresponding structures to improve the accuracy of differential diagnosis and intervention procedures for CECS.

## References

[1] Garrido F. V., Muñoz F. M. (2015). *Advanced Techniques in Musculoskeletal Medicine and Physiotherapy: Using Minimally Invasive Therapies in Practice*.

[2] Reichert B., Stelzenmueller W. (2011). *Palpation Techniques: Surface Anatomy for Physical Therapists*.

[3] Biel A. (2010). *Trail Guide to the Body*.

[4] Moore K. L., Dalley A. F., Agur A. M. (2013). *Clinically Oriented Anatomy*.

[5] Dutton M. (2004). *Orthopaedic Examination, Evaluation & Intervention*.

[6] Chaitow L. (2010). *Modern Neuromuscular Techniques*.

[7] Saunders S., Longworth S. (2006). *Injection Techniques in Orthopaedic and Sports Medicine: A Practical Manual for Doctors and Physiotherapists*.

[8] Doklamyai P., Agthong S., Chentanez V. (2008). Anatomy of the lateral femoral cutaneous nerve related to inguinal ligament, adjacent bony landmarks, and femoral artery. *Clinical Anatomy*.

[9] Coyle M., Aird M., Cobbin D. M., Zaslawski C. (2000). The cun measurement system: an investigation into its suitability in current practice. *Acupuncture in Medicine*.

[10] Drake R., Vogl A. W., Mitchell A. W. (2014). *Gray's Anatomy for Students*.

[11] Donatelli R. A., Wooden M. J. (2009). *Orthopaedic Physical Therapy*.

[12] Dunn J. C., Waterman B. R. (2014). Chronic exertional compartment syndrome of the leg in the military. *Clinics in Sports Medicine*.

[13] Ebraheim N. A., Lu J., Hao Y., Biyani A., Yeasting R. A. (1998). Anterior tibial artery and its actual projection on the lateral aspect of the tibia: A Cadaveric Study. *Surgical and Radiologic Anatomy*.

[14] Heidari N., Lidder S., Grechenig W., Tesch N. P., Weinberg A. M. (2013). The risk of injury to the anterior tibial artery in the posterolateral approach to the tibia plateau: a cadaver study. *Journal of Orthopaedic Trauma*.

[15] Liu H., Fletcher J., Dailey M., Persson M. (2009). Surface projection of the radial tunnel under the supinator muscle: a cadaver study. *Advances in Physiotherapy*.

[16] Churchill R. S., Brems J. J., Kotschi H. (2001). Glenoid size, inclination, and version: an anatomic study. *Journal of Shoulder and Elbow Surgery*.

[17] Piponov H. I., Savin D., Shah N. (2016). Glenoid version and size: does gender, ethnicity, or body size play a role?. *International Orthopaedics*.

[18] Everhart J. S., Flanigan D. C., Chaudhari A. M., Siston R. A. (2016). Tibiofemoral joint subchondral surface conformity: individual variability with race and sex-specific trends. *The Knee*.

